# Supplementary material for: Management and outcomes of myelomeningocele-associated hydrocephalus in low-income and middle-income countries: a systematic review and meta-analysis protocol
Source: BMJ Open. 2023 Feb 7;13(2):e066339. doi: 10.1136/bmjopen-2022-066339 (PMC9906258; doi:10.1136/bmjopen-2022-066339)
Supplement: Author's manuscript [file bmjopen-2022-066339.draft_revisions.pdf]

BMJ Open is committed to open peer review. As part of this commitment we make the peer review history of every article we publish publicly available.

When an article is published we post the peer reviewers' comments and the authors' responses online. We also post the versions of the paper that were used during peer review. These are the versions that the peer review comments apply to.

The versions of the paper that follow are the versions that were submitted during the peer review process. They are not the versions of record or the final published versions. They should not be cited or distributed as the published version of this manuscript.

BMJ Open is an open access journal and the full, final, typeset and author-corrected version of record of the manuscript is available on our site with no access controls, subscription charges or pay-per-view fees (http://bmjopen.bmj.com).

If you have any questions on BMJ Open's open peer review process please email info.bmjopen@bmj.com

BMJ Open

Management and outcomes of myelomeningocele-associated hydrocephalus in low- and middle-income countries: a systematic review and meta-analysis protocol

Journal:	*BMJ Open*
Manuscript ID	bmjopen-2022-066339
Article Type:	Protocol
Date Submitted by the Author:	04-Jul-2022
Complete List of Authors:	Takoutsing, Berjo Dongmo; Association of Future African Neurosurgeons<br>Yanez Touzet, Alvaro; The University of Manchester<br>Park, Jay; The University of Edinburgh Edinburgh Medical School<br>Lee, Seong Hoon ; NHS Grampian<br>Bligh, Emily; Institute of Neurological Sciences, Department of Neurosurgery<br>Egiz, Abdullah; University of Central Lancashire<br>Gillespie, Conor; University of Liverpool School of Medicine<br>Figaji, Anthony; University of Cape Town<br>Interest Group, Neurology and Neurosurgery; Neurology and Neurosurgery Interest Group
Keywords:	Paediatric neurosurgery < PAEDIATRIC SURGERY, NEUROSURGERY, NEUROLOGY

SCHOLARONE™
Manuscripts

Management and outcomes of myelomeningocele-associated hydrocephalus in low- and middle-income countries: a systematic review and meta-analysis protocol

Berjo Dongmo Takoutsing[1-2]*, Alvaro Yanez Touzet[3]*, Jay J. Park[4], Seong Hoon Lee[5], Emily R. Bligh[6,7], Abdullah Egiz[8], Conor S. Gillespie[9], Anthony Figaji[10-11], On behalf of the Neurology and Neurosurgery Interest Group

[1]Faculty of Health Sciences, University of Buea, Buea, Cameroon

[2]Research Department, Association of Future African Neurosurgeons, Yaounde, Cameroon

[3]School of Medical Sciences, Faculty of Biology, Medicine and Health, University of Manchester, Manchester, United Kingdom

[4]Edinburgh Medical School, University of Edinburgh, Edinburgh, United Kingdom

[5]Academic Critical Care & Neurosurgery, Aberdeen Royal Infirmary, Aberdeen, United Kingdom

[6]Surgical Specialties, The Queen Elizabeth University Hospital, Glasgow, United Kingdom

[7]Academic Foundation Programme, The University of Glasgow, Glasgow, United Kingdom

[8]School of Medicine, University of Central Lancashire, Preston, United Kingdom

[9]Institute of Systems, Molecular and Integrative Biology, University of Liverpool, Liverpool, United Kingdom

[10]Division of Paediatric Neuroscience (Neurosurgery), School of Child and Adolescent Health, University of Cape Town, Cape Town, South Africa

[11]Red Cross Children's Hospital, Klipfontein Road, 7700 Rondebosch, Cape Town, South Africa

Correspondence to: Alvaro Yanez Touzet; alvaro.yaneztouzet@student.manchester.ac.uk

*These authors contributed equally.

PROSPERO registration number: CRD42021285850

Word count: 1,752

For peer review only

ABSTRACT

Introduction: Hydrocephalus and myelomeningocele (MMC) place disproportionate burdens of disease on low and middle-income countries (LMICs). MMC-associated hydrocephalus and its sequelae result in a spectrum of severely devastating clinical manifestations, for which LMICs are disproportionately unprepared in terms of human, capital, and technological resources. This study aims to review and compare the management and outcomes of infant MMC-associated hydrocephalus in LMICs and high-income countries.

Methods and analysis: This systematic review and meta-analysis will follow the PRISMA 2020 guidelines. The following databases will be searched without restrictions on language, publication date, or country of origin: EMBASE, MEDLINE, The Cochrane Library, Global Index Medicus, African Journals Online, and SciELO. All peer-reviewed studies of primary data reporting management and outcomes of infant MMC-associated hydrocephalus will be included. Where high-quality homogeneous studies exist, meta-analyses will be conducted to compare the management and outcomes of MMC-associated hydrocephalus across socioeconomic and geographic regions of the world. The primary outcome will be treatment failure of the first-line hydrocephalus treatment, which we defined operationally as the performance of a second intervention for the same reason as the first. Secondary outcomes include time to failure, rates of mortality, and postoperative complications.

Ethics and dissemination: Ethical approval was not applicable because this study does not involve human participants. Dissemination strategies will include publication in a peer-reviewed journal, oral and poster presentations at conferences, and an interactive web application to facilitate interaction with the findings and promote the discussion and sharing of findings on social media.

PROSPERO registration number: CRD42021285850

Word count: 245

For peer review only

ARTICLE SUMMARY

Strengths and limitations of this study

- This review focuses on multiple treatment modes of a well-defined disease population.
- Six electronic databases that are commonly used across both high- and low-income countries will be searched.
- No restrictions on language, location, or publication date were placed during screening.
- Unpublished studies will not be sought.
- Only one operational definition of treatment failure—'the performance of a second intervention for the same reason as the first'—will be sought.
- An interactive web application dashboard will be developed to facilitate the transparent interaction with our methods and findings and promote scientific discussion and scrutiny.

Keywords: Systematic Review; Meta-Analysis; Hydrocephalus; Meningomyelocele; Infant; Developing Countries; Outcome and Process Assessment, Health Care

For peer review only

INTRODUCTION

Hydrocephalus places a disproportionate burden of disease on low and middle-income countries (LMICs) (1, 2). It affects approximately 1 in 1,000 infants worldwide, but its incidence may exceed 200,000 cases per year in developing regions like sub-Saharan Africa (3). Myelomeningocele (i.e., meningomyelocele or open spina bifida; MMC) is a common and severe spinal aetiology and constitutes a significant proportion of this population (4-6). Its prevalence also varies by geography but approximates 113 cases per 100,000 births in LMICs (7, 8), reaching 77–610 and 700 cases per 100,000 births in South Africa and Nigeria, respectively. These disorders result in a spectrum of clinical manifestations, among which MMC-associated hydrocephalus is one of the most common and debilitating (4, 9, 10).

In LMICs, the incidence of MMC-associated hydrocephalus is high and can affect as many as 75% of cases (11, 12). Times to diagnoses and treatment are often delayed in these settings and, if treatment is not promptly initiated, most patients do not survive beyond infancy (4). Treatment may involve surgery, which is prone to significant morbidity and mortality, particularly in low-resource settings (1, 3). Intervening at the earliest stage with the best possible treatment remains, therefore, a crucial step in the management of MMC-associated hydrocephalus.

Ventriculoperitoneal (VP) shunts are the current standard of treatment and are, often, the only treatment available in LMICs (1). Increasingly, patients in high-income countries are being treated with endoscopic third ventriculostomy (ETV) or combinations of ETV and choroid plexus cauterization (CPC), whose success rates and perceptions are variable (1, 3, 4, 9). The uptake, time course, and success of these techniques in LMICs are, nonetheless, less clear but amenable to aggregation and review of the global literature.

STUDY AIMS AND OBJECTIVES

Aim

This study aims to review and compare the management and outcomes of MMC-associated hydrocephalus in infants across countries and treatment modes.

Objectives

For peer review only

To review and compare:

1. The first- and second-line use of VP shunts, combinations of ETV and CPC, and conservative management;
2. The rates and times to treatment failure of VP shunts, combinations of ETV and CPC, and conservative management; and
3. Measures of mortality and postoperative complications of VP shunts, combinations of ETV and CPC, and conservative management.

Review questions

Primary question:

1. What are the rates of failure of VP shunts, ETV, combinations of ETV and CPC, and conservative treatments in infant MMC-associated hydrocephalus?

Secondary questions:

2. What are the most frequent first- and second-line treatments in the management of infant MMC-associated hydrocephalus?
3. What is the time to failure of VP shunts, ETV, combinations of ETV and CPC, and conservative treatments in infant MMC-associated hydrocephalus?
4. What are the mortality and postoperative complication rates of VP shunts, ETV, combinations of ETV and CPC, and conservative treatments in infant MMC-associated hydrocephalus?
5. Are the management and outcomes of the MMC associated with the failure of the hydrocephalus treatment?

Subgroup analyses by country and treatment mode are planned (further details in **Methods** section). The primary outcome will be the rate of treatment failure of the first-line hydrocephalus treatment. Secondary outcomes include: time to failure, rates of mortality, and postoperative complications. Operational definitions of outcomes are provided in the **Methods**.

Due to its historical standing as the local standard of treatment, our principal hypothesis is that:

H_1: VP shunts have the lowest rates of treatment failure in the infant MMC-associated hydrocephalus population.

For peer review only

Secondary hypotheses include:

H_2: VP shunts are the most frequently used first-line treatment and have the lowest mortality and complication rates.

H_3: Resource constrained environments are associated with late/worse MMC presentation amongst infants

H_4: Late/worse MMC presentation is associated with high MMC treatment failure, mortality, and complication rates.

H_5: MMC complication rates are associated with hydrocephalus treatment failure, mortality, and complication rates.

METHODS

This systematic review will be conducted following the guidelines outlined by the Preferred Reporting Items for Systematic Reviews and Meta-Analyses (PRISMA) 2020 statement (13).

Search

A search strategy was developed to identify research articles on MMC-associated hydrocephalus. This was adapted from McCarthy et al., 2019, and consisted of synonyms of 'hydrocephalus' and 'myelomeningocele' (**Supplementary Table 1**). The search was run on the following electronic databases, from inception until 5 October 2021: EMBASE, MEDLINE, The Cochrane Library, Global Index Medicus, African Journals Online, and ScieLO. No restrictions on language, location, or publication date were placed, and unpublished studies will not be sought. This search will be re-run prior to final analyses.

Study selection

Types of studies

We will include articles published in peer-reviewed journals with any of the following designs: original research, trials, cross-sectional and cohort studies, multiple case reports (i.e., >1 case), and case series. Opinion pieces, comments, letters, guidelines, editorials, single-case reports, reviews, meta-analyses, and qualitative studies will be excluded, as well as articles published in non-peer-reviewed journals.

For peer review only

Country income level

We will include studies whose data were collected in low-, lower-middle-, upper-middle-, and high-income economies, according to the World Bank Country and Lending Groups (14). Studies from high-income economies will be used as comparators. Studies whose data cannot be traced to one particular country will be excluded.

Types of participants

All studies of infants aged 2 years or younger with a clinical or imaging diagnosis of MMC-associated hydrocephalus will be included. Non-infants (i.e., >2 years) will be excluded, as will infants with diagnoses of lipomyelomeningocoele, spina bifida occulta, or unspecified spina bifida, regardless of association with hydrocephalus.

Types of interventions

Studies of infants who have undergone either conservative (non-surgical) or surgical treatment for hydrocephalus will be included. These may include VP shunting, ETV, combined ETV and CPC, combined VP shunting and ETV, and combined VP shunting, ETV, and CPC. Studies of infants who underwent treatment for MMC, but not hydrocephalus, will be excluded.

Types of outcome measures

We will include studies of primary data reporting measures of treatment failure. In this review, treatment failure will be operationally defined as the performance of a second intervention for the same reason as the first. Studies reporting measures of mortality, morbidity, postoperative complications, and follow-up duration will also be included for secondary analysis. Post-operative complications will be defined as the unfavourable result of an intervention that did not result in treatment failure, and mortality as either ‘intra-operative’, if death occurred during a procedure, or ‘perioperative’, if it occurred within 30 days of surgery. For conservative treatments, mortality will encompass deaths that occurred within 30 days of administration.

Outcomes

For peer review only

The primary outcome of our study will be the rate of treatment failure for the first-line hydrocephalus treatment. Secondary outcomes will include time to failure, rates of mortality, and postoperative complications.

Study selection

Search results will be uploaded to Rayyan (https://www.rayyan.ai) to facilitate de-duplication and independent, blinded screening (15). First, titles and abstracts will be screened by two independent reviewers against the inclusion criteria, and the eligibility of the selected abstracts will be determined by reading the full texts. Unless otherwise stated, conflicts will be resolved through discussion and, where consensus cannot be achieved, through arbitration by the senior author (AF).

Data extraction

Two reviewers will independently extract and check the data extracted from the included studies using a standardised extraction proforma (**Supplementary Table 2**). Any disputes will be settled by a third reviewer (BDT, AYT, or AF). Data items will include information on study and sample characteristics; vertebral level of MMC and aetiology of hydrocephalus; clinical presentation and method of diagnosis; treatment mode, timing, and follow-up; and primary and secondary outcomes. If data is insufficiently provided in the full text, we will contact the corresponding author to request for the missing information and wait up to two months for a response. Following correspondence, all available data will be reported and studies with missing data will not be eligible for inclusion in the meta-analysis. All data will be recorded and stored in a spreadsheet.

Risk of bias assessment

Included studies will be assessed for risk of bias by two independent reviewers of the extraction team. Randomised studies will be assessed using Version 2 of the Cochrane risk-of-bias tool for randomised trials (RoB 2) (16). Non-randomised studies will be assessed using the ROBINS I tool (17). Risk of bias assessment will be used during the analysis and results presented in terms of the primary and secondary objectives of this study.

Data analysis

For peer review only

Following extraction, data will be transferred to Python for analysis. Screening results will be presented using a PRISMA flow diagram. Study and sample characteristics will then be summarised using descriptive statistics; these may include: vertebral level of MMC; aetiology of hydrocephalus; method of diagnosis; treatment mode; indication for surgery (if surgical management); timing, and follow-up; and primary and secondary outcomes.

The use, failure, and mortality measures of VP shunts, combinations of ETV and CPC, and conservative management will then be meta-analysed. Although meta-analyses are planned, these will only become apparent after data extraction is complete. Publication bias will be assessed through the use of funnel plots and Egger's test (18). If asymmetry is found in the funnel plot, the Trim-and-Fill method will be used to account for any potential publication bias (19). The overall estimates and 95% confidence intervals will be obtained using random-effects models as per established methods (20). Cochran's Q test ($P < 0.10$) and the I^2 statistic will be used to assess heterogeneity among studies (21). Unless otherwise stated, the statistical significance will be set at $P < 0.05$, and comparisons to high-income economies will be drawn.

Mortality, failure, and complication rates will however vary with hydrocephalus aetiology, age, mode of treatment, characteristics of MMC, and socioeconomic region. Therefore, subgroup analyses will be sought, as appropriate. A combination of dichotomous, proportion, continuous, O–E, and variance meta-analyses are planned for this purpose, as appropriate, depending on the data. Recognising that case reports may present unusual and/or protracted outcomes, and randomised trials may assess active interventions improving outcomes, analyses by study design are also planned.

Strength of body of evidence

The confidence in cumulative evidence included in this review will be assessed using the GRADE approach. Two independent reviewers will use the GRADEpro Guideline Development Tool (GRADEpro GDT) to assess the quality of outcomes of this study. A third reviewer (BDT, AYT, or JJP) will settle disagreements.

Ethics and dissemination

For peer review only

Ethics approval for this study was not applicable because this study did not involve human participants. Dissemination strategies will include publication in a peer-reviewed journal, oral and poster presentations at conferences, and an interactive web application to facilitate interaction with the findings and promote the discussion and sharing of findings on social media.

Patient and public involvement

None

ACKNOWLEDGEMENTS

We thank Moniba Korch, Brian Ou Yong Ming, and Safia Farsana Shabeer for their contributions during the development and piloting of the extraction proforma.

AUTHOR CONTRIBUTIONS

BDT, AYT, and AF were responsible for conceiving the article. AF is the guarantor. BDT, AYT, JJP, and CG wrote the manuscript. AE, AF, BDT, AYT, CG, ERB, JJP, and SHL provided a critical appraisal of the manuscript. All authors critically revised and approved the final manuscript. BDT and AYT contributed equally and are joint first authors of the manuscript.

ETHICS APPROVAL

Ethics approval for this study was not applicable because this study did not involve human participants.

DATA AVAILABILITY STATEMENT

All data relevant to the study are included in the article or uploaded as supplementary information.

FUNDING

This work did not receive any funding.

COMPETING INTERESTS

None declared.

For peer review only

For peer review only

REFERENCES

1. Muir RT, Wang S, Warf BC. Global surgery for pediatric hydrocephalus in the developing world: a review of the history, challenges, and future directions. Neurosurg Focus. 2016;41(5):E11.
2. Weiss HK, Garcia RM, Omiye JA, Vervoort D, Riestenberg R, Yerneni K, et al. A Systematic Review of Neurosurgical Care in Low-Income Countries. World Neurosurg X. 2020;5:100068.
3. Kahle KT, Kulkarni AV, Limbrick DD, Jr., Warf BC. Hydrocephalus in children. Lancet. 2016;387(10020):788-99.
4. McCarthy DJ, Sheinberg DL, Luther E, McCrea HJ. Myelomeningocele-associated hydrocephalus: nationwide analysis and systematic review. Neurosurg Focus. 2019;47(4):E5.
5. Oumer M, Taye M, Aragie H, Tazebew A. Prevalence of Spina Bifida among Newborns in Africa: A Systematic Review and Meta-Analysis. Scientifica (Cairo). 2020;2020:4273510.
6. Njamnshi AK, Djientcheu Vde P, Lekoubou A, Guemse M, Obama MT, Mbu R, et al. Neural tube defects are rare among black Americans but not in sub-Saharan black Africans: the case of Yaounde - Cameroon. J Neurol Sci. 2008;270(1-2):13-7.
7. Lo A, Polšek D, Sidhu S. Estimating the burden of neural tube defects in low- and middle-income countries. J Glob Health. 2014;4(1):010402.
8. Zaganjor I, Sekkarie A, Tsang BL, Williams J, Razzaghi H, Mulinare J, et al. Describing the Prevalence of Neural Tube Defects Worldwide: A Systematic Literature Review. PLoS One. 2016;11(4):e0151586.
9. Ntimbani J, Kelly A, Lekgwara P. Myelomeningocele - A literature review. Interdisciplinary Neurosurgery. 2020;19:100502.
10. de Paul Djientcheu V, Njamnshi AK, Wonkam A, Njiki J, Guemse M, Mbu R, et al. Management of neural tube defects in a Sub-Saharan African country: the situation in Yaounde, Cameroon. J Neurol Sci. 2008;275(1-2):29-32.
11. Dewan MC, Rattani A, Mekary R, Glancz LJ, Yunusa I, Baticulon RE, et al. Global hydrocephalus epidemiology and incidence: systematic review and meta-analysis. J Neurosurg. 2018:1-15.
12. Mnguni MN, Enicker BC, Madiba TE. A perspective in the management of myelomeningocoele in the KwaZulu-Natal Province of South Africa. Childs Nerv Syst. 2020;36(7):1521-7.
13. Page MJ, McKenzie JE, Bossuyt PM, Boutron I, Hoffmann TC, Mulrow CD, et al. The PRISMA 2020 statement: an updated guideline for reporting systematic reviews. Bmj. 2021;372:n71.
14. World Bank Data Help Desk. World Bank Country and Lending Groups 2022 [Available from: https://datahelpdesk.worldbank.org/knowledgebase/articles/906519-world-bank-country-and-lending-groups.
15. Rayyan. Rayyan – Intelligent Systematic Review 2022 [Available from: https://www.rayyan.ai/.
16. Sterne JAC, Savović J, Page MJ, Elbers RG, Blencowe NS, Boutron I, et al. RoB 2: a revised tool for assessing risk of bias in randomised trials. Bmj. 2019;366:l4898.
17. Sterne JA, Hernán MA, Reeves BC, Savović J, Berkman ND, Viswanathan M, et al. ROBINS-I: a tool for assessing risk of bias in non-randomised studies of interventions. Bmj. 2016;355:i4919.

For peer review only

18. Egger M, Davey Smith G, Schneider M, Minder C. Bias in meta-analysis detected by a simple, graphical test. Bmj. 1997;315(7109):629-34.
19. Shi L, Lin L. The trim-and-fill method for publication bias: practical guidelines and recommendations based on a large database of meta-analyses. Medicine (Baltimore). 2019;98(23):e15987.
20. DerSimonian R, Laird N. Meta-analysis in clinical trials. Control Clin Trials. 1986;7(3):177-88.
21. Higgins JP, Thompson SG, Deeks JJ, Altman DG. Measuring inconsistency in meta-analyses. Bmj. 2003;327(7414):557-60.

For peer review only

SUPPLEMENTARY INFORMATION

Supplementary Table 1. Search terms for EMBASE

#	Query
1	exp hydrocephalus/ or hydrocephalus.mp.
2	(myelomeningocele or meningomyelocele).mp. or exp meningomyelocele/
3	1 and 2
4	limit 3 to human
5	limit 4 to (conference abstract or conference paper or "conference review" or editorial or erratum or letter)
6	4 not 5

For peer review only

Supplementary Table 2. Data item descriptions

Data item	Field type	Description
Study characteristics		
Study ID	Dropdown	Unique ID associated with each included article
Title	Lookup	Article title, extracted from citation
Author	Lookup	Article author(s), extracted from citation
Year of publication	Date	Only year
Year of data collection	Date	Only year
Original language of article	Dropdown	
Country of the study	Dropdown	The country where the management was done, not the country where the first author is located. From World Bank Country and Lending Groups. If management was done in multiple countries, a separate entry will be provided for each country.
Income level of study setting	Lookup	Lookup in World Bank Country and Lending Groups using **Country of the Study**
Region of study setting	Lookup	Lookup in United Nations Geoscheme using **Country of the Study**
Study design	Dropdown	Select one from: RCT, Multiple case report, Case series, Cohort study, Cross sectional study, Retrospective study (unspecified), Prospective study (unspecified), or Other (Add as comment)
Number of infants with MMC-associated HC	Integer	This need not necessarily be the same as the sample size of the study (e.g., studies looking at non-MMC aetiologies of HC)

For peer review only

<table>
<tr><td>Age
(Mean, median, standard deviation and/or range)</td><td>Integers</td><td>In days. If multiple measures of central tendency or deviation present, extract all. If there are subgroups whose ages are within two years of birth, extract data for those subgroups. Do not extract data for any subgroups outside our age range. Regex validation string for range only:
^(\d|\d\d|[1-6]\d\d|7[0-2]\d|73[0-1])\.?(\d|\d\d)?-(\d|\d\d|[1-6]\d\d|7[0-2]\d|73[0-1])\.?(\d|\d\d)?$</td></tr>
<tr><td>Sex
(Female, male)</td><td>Integer</td><td>Of Number of infants with MMCaH.</td></tr>
<tr><td colspan="3">MMC management and outcomes</td></tr>
<tr><td>Signs or symptoms of MMC</td><td>Validated free text</td><td>Of Number of infants with MMCaH. Regex validation string:
^(.)+=(\s)?(\d|\d\d|\d\d\d|\d\d\d\d)((,(.)+=(\s)?(\d|\d\d|\d\d\d|\d\d\d\d))+)?$</td></tr>
<tr><td>Vertebral level of MMC
(Cervical, thoracic, thoracolumbar, lumbar, lumbosacral, sacral)</td><td>Integer</td><td>Of Number of infants with MMCaH. Compute as raw count if not given as such (e.g., if given as a proportion or percentage). Do not double count infants across vertebral levels.</td></tr>
<tr><td>Timing of MMC closure</td><td>Dropdown</td><td>Select one from: Ante-natal or post-natal. If mixed cohort, a separate entry will be provided for each closure.</td></tr>
<tr><td>MMC closure procedure and number of patients</td><td>Validated free text</td><td>The name of the intervention and the number of patients who had that intervention. Regex validation string:
^(.)+=(\s)?(\d|\d\d|\d\d\d|\d\d\d\d)((,(.)+=(\s)?(\d|\d\d|\d\d\d|\d\d\d\d))+)?$</td></tr>
</table>

For peer review only

Timing of MMC closure after birth	Validated free text	Groups/subgroups as reported by the authors. Regex validation string: ^(.)+=(\s)?(\d|\d\d|\d\d\d|\d\d\d\d)((,(.)+=(\s)?(\d|\d\d|\d\d\d|\d\d\d\d))+)?$
Age at MMC closure (Mean, median, standard deviation and/or range)	Integers	As in **Age.** Regex validation string for **range** only: ^(\d|\d\d|\d\d\d|\d\d\d\d|\d\d\d\d\d)\.?(\d|\d\d)?-(\d|\d\d|\d\d\d|\d\d\d\d|\d\d\d\d\d)\.?(\d|\d\d)?$
Intra-operative mortality of MMC closure	Integer	Death during procedure. Compute as raw count if not given as such (e.g., if given as a proportion or percentage).
Peri-operative mortality of MMC closure	Integer	Death within 30 days of a procedure. Compute as raw count if not given as such (e.g., if given as a proportion or percentage).
Post-operative complications of MMC closure	Integer	Raw counts for Wound breakdown, Wound infection, and/or Meningitis. Regex validation string for **miscellaneous** only: ^(.)+=(\s)?(\d|\d\d|\d\d\d|\d\d\d\d)((,(.)+=(\s)?(\d|\d\d|\d\d\d|\d\d\d\d))+)?$
First line intervention for HC		
Patient sample	Dropdown	Select from: Selected or Unselected.
Age at diagnosis of HC (Pre-natal or post-natal)	Integers	As in **Age.**
Method of diagnosis of HC (CT imaging, MRI imaging, Ultrasonography, Clinical manifestation only, and Miscellaneous)	Integers	Raw counts for number of infants diagnosed with these methods. Regex validation string for **miscellaneous** only: ^(.)+=(\s)?(\d|\d\d|\d\d\d|\d\d\d\d)((,(.)+=(\s)?(\d|\d\d|\d\d\d|\d\d\d\d))+)?$

For peer review only

<table>
<tr><td>Indications for HC surgery
(Full or bulging fontanelle, Increased head circumference, CSF leaking from the wound, Symptoms of raised pressure, Other indications)</td><td>Integers</td><td>Raw counts for number of infants with these indications for HC surgery. Symptoms of raised pressure may include: These include: prominent scalp veins, high pitched cry, irritability, seizure, poor feeding, developmental delay, vomiting, setting sun sign, head suture and diastasis. Regex validation string for other indications only:
^(.)+=(\s)?(\d|\d\d|\d\d\d|\d\d\d\d)((,(.)+=(\s)?(\d|\d\d|\d\d\d|\d\d\d\d))+)?$</td></tr>
<tr><td>Co-morbidities
(Congenital heart defects, Congenital gastrointestinal malformations, Congenital face and neck malformations, Miscellaneous)</td><td>Integers</td><td>Raw counts for number of infants with these co-morbidities. Co-morbidities may not be recorded here if they are consequences of MMC/HC. Regex validation string for miscellaneous only:
^(.)+=(\s)?(\d|\d\d|\d\d\d|\d\d\d\d)((,(.)+=(\s)?(\d|\d\d|\d\d\d|\d\d\d\d))+)?$</td></tr>
<tr><td>Exacerbating factors
(Meningitis, Ventriculitis or Any other brain infection)</td><td>Integers</td><td>Raw counts for number of infants who had either meningitis or ventriculitis or any other brain infection before the HC.</td></tr>
<tr><td>Temporising procedures
(Subgaleal shunting or EVD)</td><td>Integers</td><td>Raw counts for number of infants who underwent subgaleal shunting or EVD as temporising procedures.</td></tr>
<tr><td>1st-line intervention for HC
(Conservative, VP shunting, ETV, Combined ETV and CPC, Failed ETV that was converted to VP shunt, or Miscellaneous)</td><td>Integers</td><td>Raw counts for number of infants who underwent one of these procedures as first-line treatment for HC. Non-surgical treatment is interpreted as conservative. If the infant underwent both VP and ETV, determine which was the primary one. For an infant to be recorded as a failed ETV that was converted to VP shunt,</td></tr>
</table>

For peer review only

<table>
<tr><td></td><td></td><td>the procedures must have happened in one sitting; alternatively, determine which was the primary one. Regex validation string for miscellaneous only: ^(.)+=(\s)?(\d|\d\d|\d\d\d|\d\d\d\d)((,(.)+=(\s)?(\d|\d\d|\d\d\d|\d\d\d\d))+)?$</td></tr>
<tr><td>Age at first-line procedure</td><td>Integers</td><td>As in Age.</td></tr>
<tr><td>Follow-up period</td><td>Integer</td><td>In weeks</td></tr>
<tr><td>Intra-operative mortality of 1st-line HC procedure</td><td>Integer</td><td>As in Intra-operative mortality of MMC closure</td></tr>
<tr><td>Peri-operative mortality of 1st-line HC procedure</td><td>Integer</td><td>As in Peri-operative mortality of MMC closure</td></tr>
<tr><td>Post-operative complications of 1st-line HC procedure (Wound, Procedure failure, Wound infection, Wound dehiscence, Meningitis, Ventriculitis, CSF leakage, Focal Neurological deficits, Overdrainage, Intracranial haemorrhage, Any biochemical disturbance, and Any other complications)</td><td>Integers</td><td>Raw counts for Wound, Procedure failure, Wound infection, Wound dehiscence, Meningitis, Ventriculitis, CSF leakage, Focal Neurological deficits, Overdrainage, Intracranial haemorrhage, Any biochemical disturbance, and Any other complications. Regex validation string for any biochemical disturbance and any other complications: ^(.)+=(\s)?(\d|\d\d|\d\d\d|\d\d\d\d)((,(.)+=(\s)?(\d|\d\d|\d\d\d|\d\d\d\d))+)?$</td></tr>
<tr><td>Number of procedures per patient</td><td>Integers</td><td>Raw counts.</td></tr>
</table>

For peer review only

(Mean, Median, Standard deviation, and/or Range)		
Second line intervention for HC		
Time to 1st procedure failure, if any (Mean, Median, Standard deviation and/or Range)	Integers	The time between 1st-line HC procedure and 2nd-line HC procedure. In weeks.
Any information on why the treatment failed	Free text	
2nd-line HC procedure	Integers	As in **1st-line HC procedure**
Age at second-line procedure	Integers	As in **Age at first-line procedure;** except that in weeks.
Intra-operative mortality of 2nd-line HC procedure	Integer	As in **Intra-operative mortality of 1st line HC procedure**
Peri-operative mortality of 2nd-line HC procedure	Integer	As in **Peri-operative mortality of 1st line HC procedure**
Post-operative complications of 2nd-line HC procedure	Integers	As in **Post-operative complications of 1st-line HC procedure**
Any further procedures for HC (repeat data items in **Second line intervention for HC** subheading)		
Overall outcomes		

For peer review only

Overall complication rate, if any (Mean, Median, Standard deviation, and/or Range)	Integers	Number of infants with any complications divided by the number of infants that underwent any surgery
Overall incidence rate, if any (Mean, Median, Standard deviation, and/or Range)	Integers	Number of procedure failures divided by the number of procedures. Also known as the overall failure rate

CPC: Choroid plexus cauterisation, ETV: Endoscopic third ventriculostomy, EVD: External ventricular drainage, HC: Hydrocephalus, MMC: Myelomeningocele, VP: ventriculoperitoneal

For peer review only

PRISMA-P 2015 Checklist

This checklist has been adapted for use with protocol submissions to *Systematic Reviews* from Table 3 in Moher D et al: Preferred reporting items for systematic review and meta-analysis protocols (PRISMA-P) 2015 statement. *Systematic Reviews* 2015 **4**:1

Section/topic	#	Checklist item	Information reported: Yes	Information reported: No	Line number(s)
**ADMINISTRATIVE INFORMATION**					
**Title**					
Identification	1a	Identify the report as a protocol of a systematic review	☑	☐	
Update	1b	If the protocol is for an update of a previous systematic review, identify as such	☐	☑	
**Registration**	2	If registered, provide the name of the registry (e.g., PROSPERO) and registration number in the Abstract	☑	☐	
**Authors**					
Contact	3a	Provide name, institutional affiliation, and e-mail address of all protocol authors; provide physical mailing address of corresponding author	☑	☐	
Contributions	3b	Describe contributions of protocol authors and identify the guarantor of the review	☑	☐	
**Amendments**	4	If the protocol represents an amendment of a previously completed or published protocol, identify as such and list changes; otherwise, state plan for documenting important protocol amendments	☐	☑	
**Support**					
Sources	5a	Indicate sources of financial or other support for the review	☑	☐	
Sponsor	5b	Provide name for the review funder and/or sponsor	☐	☑	
Role of sponsor/funder	5c	Describe roles of funder(s), sponsor(s), and/or institution(s), if any, in developing the protocol	☐	☑	
**INTRODUCTION**					
**Rationale**	6	Describe the rationale for the review in the context of what is already known	☑	☐	
**Objectives**	7	Provide an explicit statement of the question(s) the review will address with reference to participants, interventions, comparators, and outcomes (PICO)	☑	☐	

For peer review only

BioMed Central The Open Access Publisher

Section/topic	#	Checklist item	Information reported: Yes	Information reported: No	Line number(s)
**METHODS**					
**Eligibility criteria**	8	Specify the study characteristics (e.g., PICO, study design, setting, time frame) and report characteristics (e.g., years considered, language, publication status) to be used as criteria for eligibility for the review	✓	☐	
**Information sources**	9	Describe all intended information sources (e.g., electronic databases, contact with study authors, trial registers, or other grey literature sources) with planned dates of coverage	✓	☐	
**Search strategy**	10	Present draft of search strategy to be used for at least one electronic database, including planned limits, such that it could be repeated	✓	☐	
STUDY RECORDS					
Data management	11a	Describe the mechanism(s) that will be used to manage records and data throughout the review	☐	☐	
Selection process	11b	State the process that will be used for selecting studies (e.g., two independent reviewers) through each phase of the review (i.e., screening, eligibility, and inclusion in meta-analysis)	✓	☐	
Data collection process	11c	Describe planned method of extracting data from reports (e.g., piloting forms, done independently, in duplicate), any processes for obtaining and confirming data from investigators	✓	☐	
**Data items**	12	List and define all variables for which data will be sought (e.g., PICO items, funding sources), any pre-planned data assumptions and simplifications	☐	☐	
**Outcomes and prioritization**	13	List and define all outcomes for which data will be sought, including prioritization of main and additional outcomes, with rationale	✓	☐	
**Risk of bias in individual studies**	14	Describe anticipated methods for assessing risk of bias of individual studies, including whether this will be done at the outcome or study level, or both; state how this information will be used in data synthesis	✓	☐	
DATA					
**Synthesis**	15a	Describe criteria under which study data will be quantitatively synthesized	✓	☐	
	15b	If data are appropriate for quantitative synthesis, describe planned summary measures, methods of handling data, and methods of combining data from studies, including any planned exploration of consistency (e.g., I^2, Kendall's tau)	✓	☐	
	15c	Describe any proposed additional analyses (e.g., sensitivity or subgroup analyses, meta-regression)	✓	☐	
	15d	If quantitative synthesis is not appropriate, describe the type of summary planned	☐	✓	
**Meta-bias(es)**	16	Specify any planned assessment of meta-bias(es) (e.g., publication bias across studies, selective	✓	☐	

For peer review only

BioMed Central The Open Access Publisher

Section/topic	#	Checklist item	Information reported		Line number(s)
			Yes	No	
		reporting within studies)			
**Confidence in cumulative evidence**	17	Describe how the strength of the body of evidence will be assessed (e.g., GRADE)	☑	☐	

For peer review only

BioMed Central
The Open Access Publisher

Management and outcomes of myelomeningocele-associated hydrocephalus in low- and middle-income countries: a systematic review and meta-analysis protocol

Journal:	*BMJ Open*
Manuscript ID	bmjopen-2022-066339.R1
Article Type:	Protocol
Date Submitted by the Author:	06-Dec-2022

Complete List of Authors:	Takoutsing, Berjo Dongmo; Association of Future African Neurosurgeons<br>Yanez Touzet, Alvaro; The University of Manchester<br>Park, Jay; The University of Edinburgh Edinburgh Medical School<br>Lee, Seong Hoon ; NHS Grampian<br>Bligh, Emily; Institute of Neurological Sciences, Department of Neurosurgery<br>Egiz, Abdullah; University of Central Lancashire<br>Gillespie, Conor; University of Liverpool School of Medicine<br>Figaji, Anthony; University of Cape Town<br>Interest Group, Neurology and Neurosurgery; Neurology and Neurosurgery Interest Group

**Primary Subject Heading**:	Neurology
Secondary Subject Heading:	Surgery, Global health
Keywords:	Paediatric neurosurgery < PAEDIATRIC SURGERY, NEUROSURGERY, NEUROLOGY

SCHOLARONE™
Manuscripts

Management and outcomes of myelomeningocele-associated hydrocephalus in low- and middle-income countries: a systematic review and meta-analysis protocol

Berjo Dongmo Takoutsing[1-2]*, Alvaro Yanez Touzet[3]*, Jay J. Park[4], Seong Hoon Lee[5], Emily R. Bligh[6,7], Abdullah Egiz[8], Conor S. Gillespie[9], Anthony Figaji[10-11], On behalf of the Neurology and Neurosurgery Interest Group

[1]Faculty of Health Sciences, University of Buea, Buea, Cameroon

[2]Research Department, Association of Future African Neurosurgeons, Yaounde, Cameroon

[3]School of Medical Sciences, Faculty of Biology, Medicine and Health, University of Manchester, Manchester, United Kingdom

[4]Edinburgh Medical School, University of Edinburgh, Edinburgh, United Kingdom

[5]Academic Critical Care & Neurosurgery, Aberdeen Royal Infirmary, Aberdeen, United Kingdom

[6]Surgical Specialties, The Queen Elizabeth University Hospital, Glasgow, United Kingdom

[7]Academic Foundation Programme, The University of Glasgow, Glasgow, United Kingdom

[8]School of Medicine, University of Central Lancashire, Preston, United Kingdom

[9]Institute of Systems, Molecular and Integrative Biology, University of Liverpool, Liverpool, United Kingdom

[10]Division of Paediatric Neuroscience (Neurosurgery), School of Child and Adolescent Health, University of Cape Town, Cape Town, South Africa

[11]Red Cross Children's Hospital, Klipfontein Road, 7700 Rondebosch, Cape Town, South Africa

Correspondence to: Alvaro Yanez Touzet; alvaro.yaneztouzet@student.manchester.ac.uk

*These authors contributed equally.

PROSPERO registration number: CRD42021285850

Word count: 1,860

For peer review only - http://bmjopen.bmj.com/site/about/guidelines.xhtml

ABSTRACT

Introduction: Hydrocephalus and myelomeningocele (MMC) place disproportionate burdens of disease on low and middle-income countries (LMICs). MMC-associated hydrocephalus and its sequelae result in a spectrum of severely devastating clinical manifestations, for which LMICs are disproportionately unprepared in terms of human, capital, and technological resources. This study aims to review and compare the management and outcomes of infant MMC-associated hydrocephalus in LMICs and high-income countries.

Methods and analysis: This systematic review and meta-analysis will follow the PRISMA 2020 guidelines. The following databases will be searched without restrictions on language, publication date, or country of origin: EMBASE, MEDLINE, The Cochrane Library, Global Index Medicus, African Journals Online, and SciELO. All peer-reviewed studies of primary data reporting management and outcomes of infant MMC-associated hydrocephalus will be included. Where high-quality homogeneous studies exist, meta-analyses will be conducted to compare the management and outcomes of MMC-associated hydrocephalus across socioeconomic and geographic regions of the world. The primary outcome will be treatment failure of the first-line hydrocephalus treatment, which we defined operationally as the performance of a second intervention for the same reason as the first. Secondary outcomes include time to failure, rates of mortality, and postoperative complications.

Ethics and dissemination: Ethical approval was not applicable because this study does not involve human participants. Dissemination strategies will include publication in a peer-reviewed journal, oral and poster presentations at conferences, and an interactive web application to facilitate interaction with the findings and promote the discussion and sharing of findings on social media.

PROSPERO registration number: CRD42021285850

Word count: 245

For peer review only

ARTICLE SUMMARY

Strengths and limitations of this study

- This review focuses on multiple treatment modes of a well-defined disease population.
- Six electronic databases that are commonly used across both high- and low-income countries will be searched without restrictions on language, location, or publication date.
- The representativeness of the sample will rely on the quality of reporting of myelomeningocoele-associated hydrocephalus in the literature.
- Only one operational definition of treatment failure—'the performance of a second intervention for the same reason as the first'—will be sought.
- An interactive web application dashboard will be developed to facilitate the transparent interaction with our methods and findings and promote scientific discussion and scrutiny.

Keywords: Systematic Review; Meta-Analysis; Hydrocephalus; Meningomyelocele; Infant; Developing Countries; Outcome and Process Assessment, Health Care

For peer review only

For peer review only - http://bmjopen.bmj.com/site/about/guidelines.xhtml

INTRODUCTION

Hydrocephalus places a disproportionate burden of disease on low- and middle-income countries (LMICs) (1, 2). It affects approximately 1 in 1,000 infants worldwide, but its incidence may exceed 200,000 cases per year in developing regions like sub-Saharan Africa (3). Myelomeningocele (i.e., meningomyelocele or open spina bifida; MMC) is a common and severe spinal aetiology and constitutes a significant proportion of this population (4-6). Its prevalence also varies by geography but approximates 113 cases per 100,000 births in LMICs (7, 8), reaching 77–610 and 700 cases per 100,000 births in South Africa and Nigeria, respectively. These disorders result in a spectrum of clinical manifestations, among which MMC-associated hydrocephalus is one of the most common and debilitating (4, 9, 10).

In LMICs, the incidence of MMC-associated hydrocephalus is high and can affect as many as 75% of cases (11, 12). Times to diagnoses and treatment are often delayed in these settings and, if treatment is not promptly initiated, most patients do not survive beyond infancy (4). Treatment may involve surgery, which is prone to significant morbidity and mortality, particularly in low-resource settings (1, 3). Intervening at the earliest stage with the best possible treatment remains, therefore, a crucial step in the management of MMC-associated hydrocephalus.

Ventriculoperitoneal (VP) shunts are the current standard of treatment in LMICs (1). Increasingly, patients are being treated with endoscopic third ventriculostomy (ETV) or combinations of ETV and choroid plexus cauterization (CPC), whose success rates and perceptions are variable (1, 3, 4, 9). Although ETV/CPCs were first developed and validated in LMICs as the primary management for MMC-associated hydrocephalus, there remains limited aggregated data regarding their outcomes in the very countries these procedures were developed in. As such, the practice and outcomes of these techniques in LMICs warrant systematic review and meta-analysis. Previous work has examined the management and outcomes of hydrocephalus in HICs and LMICs (13, 14) albeit in unstratified medical subject headings (MeSH) age groups and aetiologies.

STUDY AIMS AND OBJECTIVES

Aim

For peer review only

This study aims to review and compare the management and outcomes of MMC-associated hydrocephalus in infants across countries and treatment modes.

Objectives

To review and compare:

1. The first- and second-line use of VP shunts, combinations of ETV and CPC, and conservative management;
2. The rates and times to treatment failure of VP shunts, combinations of ETV and CPC, and conservative management; and
3. Measures of mortality and postoperative complications of VP shunts, combinations of ETV and CPC, and conservative management.

Review questions

Primary question:

1. What are the rates of failure of VP shunts, ETV, combinations of ETV and CPC, and conservative treatments in infant MMC-associated hydrocephalus?

Secondary questions:

2. What are the most frequent first- and second-line treatments in the management of infant MMC-associated hydrocephalus?
3. What is the time to failure of VP shunts, ETV, combinations of ETV and CPC, and conservative treatments in infant MMC-associated hydrocephalus?
4. What are the mortality and postoperative complication rates of VP shunts, ETV, combinations of ETV and CPC, and conservative treatments in infant MMC-associated hydrocephalus?
5. Are the management and outcomes of the MMC associated with the failure of the hydrocephalus treatment?

Subgroup analyses by country and treatment mode are planned (further details in **Methods** section). The primary outcome will be the rate of treatment failure of the first-line hydrocephalus treatment. Secondary outcomes include: time to failure, rates of mortality, and postoperative complications. Operational definitions of outcomes are provided in the **Methods**.

For peer review only

Due to its historical standing as the local standard of treatment, our principal hypothesis is that:

H_1: VP shunts have the lowest rates of treatment failure in the infant MMC-associated hydrocephalus population.

Secondary hypotheses include:

H_2: VP shunts are the most frequently used first-line treatment and have the lowest mortality and complication rates.

H_3: Resource constrained environments are associated with late/worse MMC presentation amongst infants

H_4: Late/worse MMC presentation is associated with high MMC treatment failure, mortality, and complication rates.

H_5: MMC complication rates are associated with hydrocephalus treatment failure, mortality, and complication rates.

METHODS

This systematic review will be conducted following the guidelines outlined by the Preferred Reporting Items for Systematic Reviews and Meta-Analyses (PRISMA) 2020 statement (15).

Search

A search strategy was developed to identify research articles on MMC-associated hydrocephalus. This was adapted from McCarthy et al., 2019, and consisted of synonyms of 'hydrocephalus' and 'myelomeningocele' (**Supplementary Table 1**). The search was run on the following electronic databases, from inception until 5 October 2021: EMBASE, MEDLINE, The Cochrane Library, Global Index Medicus, African Journals Online, and ScieLO. No restrictions on language, location, or publication date were placed, and unpublished studies will not be sought. Searches in all databases will be re-run prior to final analysis.

Study selection

Types of studies

We will include articles published in peer-reviewed journals with any of the following designs: original research, trials, cross-sectional and cohort studies, multiple case

For peer review only - http://bmjopen.bmj.com/site/about/guidelines.xhtml

reports (i.e., >1 case), and case series. Opinion pieces, comments, letters, guidelines, editorials, single-case reports, reviews, meta-analyses, and qualitative studies will be excluded, as well as articles published in non-peer-reviewed journals.

Country income level

We will include studies whose data were collected in low-, lower-middle-, upper-middle-, and high-income economies, according to the World Bank Country and Lending Groups (16). Studies from high-income economies will be used as comparators. Studies whose data cannot be traced to one particular country will be excluded.

Types of participants

All studies of infants aged 2 years or younger with a clinical or imaging diagnosis of MMC-associated hydrocephalus will be included. Non-infants (i.e., >2 years) will be excluded, as will infants with diagnoses of lipomyelomeningocoele, spina bifida occulta, or unspecified spina bifida, regardless of association with hydrocephalus.

Types of interventions

Studies of infants who have undergone either surgical or conservative treatment for hydrocephalus will be included. Surgical treatment includes VP shunting, ETV, combined ETV and CPC, combined VP shunting and ETV, and combined VP shunting, ETV, and CPC. We defined conservative treatment as non-surgical medical or pharmacological treatment. Studies of infants who underwent treatment for MMC, but not hydrocephalus, will be excluded, as will patients who received neither surgical nor conservative interventions.

Types of outcome measures

We will include studies of primary data reporting measures of treatment failure. In this review, treatment failure will be operationally defined as the performance of a second intervention for the same reason as the first. Studies reporting measures of mortality, morbidity, postoperative complications, and follow-up duration will also be included for secondary analysis. Post-operative complications will be defined as the unfavourable result of an intervention that did not result in treatment failure, and mortality as either ‘intra-operative’, if death occurred during a procedure, or ‘perioperative’, if it occurred

For peer review only - http://bmjopen.bmj.com/site/about/guidelines.xhtml

within 30 days of surgery. For conservative treatments, mortality will encompass deaths that occurred within 30 days of administration.

Outcomes

The primary outcome of our study will be the rate of treatment failure for the first-line hydrocephalus treatment. Secondary outcomes will include time to failure, rates of mortality, and postoperative complications.

Study selection

Search results will be uploaded to Rayyan (https://www.rayyan.ai) to facilitate de-duplication and independent, blinded screening (17). First, titles and abstracts will be screened by two independent reviewers against the inclusion criteria, and the eligibility of the selected abstracts will be determined by reading the full texts. Unless otherwise stated, conflicts will be resolved through discussion and, where consensus cannot be achieved, through arbitration by the senior author (AF).

Data extraction

Two reviewers will independently extract and check the data extracted from the included studies using a standardised extraction proforma in Google Sheets (**Supplementary Table 2**). Any disputes will be settled by a third reviewer (BDT, AYT, or AF). Data items will include information on study and sample characteristics; vertebral level of MMC and aetiology of hydrocephalus; clinical presentation and method of diagnosis; treatment mode, timing, and follow-up; and primary and secondary outcomes. If data is insufficiently provided in the full text, we will contact the corresponding author to request for the missing information and wait up to two months for a response. Following correspondence, all available data will be reported and studies with missing data will not be eligible for inclusion in the meta-analysis. All data will be recorded and stored in a spreadsheet.

Risk of bias assessment

Included studies will be assessed for risk of bias by two independent reviewers of the extraction team. Randomised studies will be assessed using Version 2 of the Cochrane risk-of-bias tool for randomised trials (RoB 2) (18). Non-randomised studies will be assessed using the ROBINS I tool (19). Risk of bias assessment will be used

For peer review only - http://bmjopen.bmj.com/site/about/guidelines.xhtml

during the analysis and results presented in terms of the primary and secondary objectives of this study.

Data analysis

Following extraction, data will be transferred to Python for analysis. Screening results will be presented using a PRISMA flow diagram. Study and sample characteristics will then be summarised using descriptive statistics; these may include: vertebral level of MMC; aetiology of hydrocephalus; method of diagnosis; treatment mode; indication for surgery (if surgical management); timing, and follow-up; and primary and secondary outcomes.

The use, failure, and mortality measures of VP shunts, combinations of ETV and CPC, and conservative management will then be meta-analysed. Although meta-analyses are planned, these will only become apparent after data extraction is complete. Publication bias will be assessed through the use of funnel plots and Egger's test (20). If asymmetry is found in the funnel plot, the Trim-and-Fill method will be used to account for any potential publication bias (21). The overall estimates and 95% confidence intervals will be obtained using random-effects models as per established methods (22). Cochran's Q test ($P < 0.10$) and the I^2 statistic will be used to assess heterogeneity among studies (23). Unless otherwise stated, the statistical significance will be set at $P < 0.05$, and comparisons to high-income economies will be drawn.

Mortality, failure, and complication rates will however vary with hydrocephalus aetiology, age, mode of treatment, characteristics of MMC, socioeconomic region and, crucially, duration of follow-up. Therefore, subgroup analyses will be sought, as appropriate, and particular care will be taken when interpreting failure between shunt placement and endoscopic treatments, owing to contrasting patterns of failure over the shorter and longer terms. A combination of dichotomous, proportion, continuous, O–E, and variance meta-analyses are planned for this purpose, as appropriate, depending on the data, and may include a potential subgroup analysis of the primary outcome into short-term rates and long-term rates. Recognising that case reports may present unusual and/or protracted outcomes, and randomised trials may assess active interventions improving outcomes, analyses by study design are also planned.

For peer review only - http://bmjopen.bmj.com/site/about/guidelines.xhtml

For peer review only

Strength of body of evidence

The confidence in cumulative evidence included in this review will be assessed using the GRADE approach. Two independent reviewers will use the GRADEpro Guideline Development Tool (GRADEpro GDT) to assess the quality of outcomes of this study. A third reviewer (BDT, AYT, or JJP) will settle disagreements.

Ethics and dissemination

Ethics approval for this study was not applicable because this study did not involve human participants. Dissemination strategies will include publication in a peer-reviewed journal, oral and poster presentations at conferences, and an interactive web application to facilitate interaction with the findings and promote the discussion and sharing of findings on social media.

Patient and public involvement

None

ACKNOWLEDGEMENTS

We thank Moniba Korch, Brian Ou Yong Ming, and Safia Farsana Shabeer for their contributions during the development and piloting of the extraction proforma.

AUTHOR CONTRIBUTIONS

BDT, AYT, and AF were responsible for conceiving the article. AF is the guarantor. BDT, AYT, JJP, and CG wrote the manuscript. AE, AF, BDT, AYT, CG, ERB, JJP, and SHL provided a critical appraisal of the manuscript. All authors critically revised and approved the final manuscript. BDT and AYT contributed equally and are joint first authors of the manuscript.

ETHICS APPROVAL

Ethics approval for this study was not applicable because this study did not involve human participants.

DATA AVAILABILITY STATEMENT

For peer review only - http://bmjopen.bmj.com/site/about/guidelines.xhtml

All data relevant to the study are included in the article or uploaded as supplementary information.

FUNDING
This work did not receive any funding.

COMPETING INTERESTS

None declared.

For peer review only

For peer review only - http://bmjopen.bmj.com/site/about/guidelines.xhtml

REFERENCES

1. Muir RT, Wang S, Warf BC. Global surgery for pediatric hydrocephalus in the developing world: a review of the history, challenges, and future directions. Neurosurg Focus. 2016;41(5):E11.
2. Weiss HK, Garcia RM, Omiye JA, Vervoort D, Riestenberg R, Yerneni K, et al. A Systematic Review of Neurosurgical Care in Low-Income Countries. World Neurosurg X. 2020;5:100068.
3. Kahle KT, Kulkarni AV, Limbrick DD, Jr., Warf BC. Hydrocephalus in children. Lancet. 2016;387(10020):788-99.
4. McCarthy DJ, Sheinberg DL, Luther E, McCrea HJ. Myelomeningocele-associated hydrocephalus: nationwide analysis and systematic review. Neurosurg Focus. 2019;47(4):E5.
5. Oumer M, Taye M, Aragie H, Tazebew A. Prevalence of Spina Bifida among Newborns in Africa: A Systematic Review and Meta-Analysis. Scientifica (Cairo). 2020;2020:4273510.
6. Njamnshi AK, Djientcheu Vde P, Lekoubou A, Guemse M, Obama MT, Mbu R, et al. Neural tube defects are rare among black Americans but not in sub-Saharan black Africans: the case of Yaounde - Cameroon. J Neurol Sci. 2008;270(1-2):13-7.
7. Lo A, Polšek D, Sidhu S. Estimating the burden of neural tube defects in low- and middle-income countries. J Glob Health. 2014;4(1):010402.
8. Zaganjor I, Sekkarie A, Tsang BL, Williams J, Razzaghi H, Mulinare J, et al. Describing the Prevalence of Neural Tube Defects Worldwide: A Systematic Literature Review. PLoS One. 2016;11(4):e0151586.
9. Ntimbani J, Kelly A, Lekgwara P. Myelomeningocele - A literature review. Interdisciplinary Neurosurgery. 2020;19:100502.
10. de Paul Djientcheu V, Njamnshi AK, Wonkam A, Njiki J, Guemse M, Mbu R, et al. Management of neural tube defects in a Sub-Saharan African country: the situation in Yaounde, Cameroon. J Neurol Sci. 2008;275(1-2):29-32.
11. Dewan MC, Rattani A, Mekary R, Glancz LJ, Yunusa I, Baticulon RE, et al. Global hydrocephalus epidemiology and incidence: systematic review and meta-analysis. J Neurosurg. 2018:1-15.
12. Mnguni MN, Enicker BC, Madiba TE. A perspective in the management of myelomeningocoele in the KwaZulu-Natal Province of South Africa. Childs Nerv Syst. 2020;36(7):1521-7.
13. Ben-Israel D, Mann JA, Yang MMH, Isaacs AM, Cadieux M, Sader N, et al. Clinical outcomes in pediatric hydrocephalus patients treated with endoscopic third ventriculostomy and choroid plexus cauterization: a systematic review and meta-analysis. J Neurosurg Pediatr. 2022:1-13.
14. Pande A, Lamba N, Mammi M, Gebrehiwet P, Trenary A, Doucette J, et al. Endoscopic third ventriculostomy versus ventriculoperitoneal shunt in pediatric and adult population: a systematic review and meta-analysis. Neurosurg Rev. 2021;44(3):1227-41.
15. Page MJ, McKenzie JE, Bossuyt PM, Boutron I, Hoffmann TC, Mulrow CD, et al. The PRISMA 2020 statement: an updated guideline for reporting systematic reviews. Bmj. 2021;372:n71.
16. World Bank Data Help Desk. World Bank Country and Lending Groups 2022 [Available from: https://datahelpdesk.worldbank.org/knowledgebase/articles/906519-world-bank-country-and-lending-groups.

For peer review only - http://bmjopen.bmj.com/site/about/guidelines.xhtml

17. Rayyan. Rayyan – Intelligent Systematic Review 2022 [Available from: https://www.rayyan.ai/.
18. Sterne JAC, Savović J, Page MJ, Elbers RG, Blencowe NS, Boutron I, et al. RoB 2: a revised tool for assessing risk of bias in randomised trials. Bmj. 2019;366:l4898.
19. Sterne JA, Hernán MA, Reeves BC, Savović J, Berkman ND, Viswanathan M, et al. ROBINS-I: a tool for assessing risk of bias in non-randomised studies of interventions. Bmj. 2016;355:i4919.
20. Egger M, Davey Smith G, Schneider M, Minder C. Bias in meta-analysis detected by a simple, graphical test. Bmj. 1997;315(7109):629-34.
21. Shi L, Lin L. The trim-and-fill method for publication bias: practical guidelines and recommendations based on a large database of meta-analyses. Medicine (Baltimore). 2019;98(23):e15987.
22. DerSimonian R, Laird N. Meta-analysis in clinical trials. Control Clin Trials. 1986;7(3):177-88.
23. Higgins JP, Thompson SG, Deeks JJ, Altman DG. Measuring inconsistency in meta-analyses. Bmj. 2003;327(7414):557-60.

For peer review only - http://bmjopen.bmj.com/site/about/guidelines.xhtml

SUPPLEMENTARY INFORMATION

Supplementary Table 1. Search terms for all databases

EMBASE

#	Query
1	exp hydrocephalus/ or hydrocephalus.mp.
2	(myelomeningocele or meningomyelocele).mp. or exp meningomyelocele/
3	1 and 2
4	limit 3 to human
5	limit 4 to (conference abstract or conference paper or “conference review” or editorial or erratum or letter)
6	4 not 5

MEDLINE

#	Query
1	exp Hydrocephalus/ or hydrocephalus.mp.
2	(myelomeningocele or meningomyelocele).mp. or exp Meningomyelocele/
3	1 and 2
4	limit 3 to humans
5	limit 4 to (conference abstract or conference paper or "conference review" or editorial or erratum or letter)
6	4 not 5

PubMed

#	Query
1	(("meningomyelocele"[MeSH Terms] OR "meningomyelocele"[All Fields] OR "myelomeningocele"[All Fields] OR "myelomeningoceles"[All Fields] OR ("meningomyelocele"[MeSH Terms] OR "meningomyelocele"[All Fields] OR "meningomyeloceles"[All Fields])) AND ("hydrocephalus"[MeSH Terms] OR "hydrocephalus"[All Fields])) AND (humans[Filter]) NOT ((booksdocs[Filter] OR editorial[Filter] OR letter[Filter] OR meta-analysis[Filter] OR review[Filter] OR systematicreview[Filter]))

For peer review only

NANSIG

The Cochrane Library

#	Query
1	hydrocephalus
2	MeSH descriptor: [Hydrocephalus] explode all trees
3	#1 or #2
4	myelomeningocele or meningomyelocele
5	MeSH descriptor: [Meningomyelocele] explode all trees
6	#4 or #5
7	#3 and #6
8	#7 in Cochrane Reviews, Cochrane Protocols, Editorials
9	#7 not #8

Global Index Medicus

#	Query
1	(myelomeningocele or meningomyelocele) and hydrocephalus

African Journals Online

#	Query
1	(myelomeningocele or meningomyelocele) and hydrocephalus

SciELO

#	Query
1	(((myelomeningocele)) OR (meningomyelocele)) AND (hydrocephalus)

For peer review only

NANSIG

Supplementary Table 2. Data item descriptions

Data item	Field type	Description
Study characteristics		
Study ID	Dropdown	Unique ID associated with each included article
Title	Lookup	Article title, extracted from citation
Author	Lookup	Article author(s), extracted from citation
Year of publication	Date	Only year
Year of data collection	Date	Only year
Original language of article	Dropdown	
Country of the study	Dropdown	The country where the management was done, not the country where the first author is located. From World Bank Country and Lending Groups. If management was done in multiple countries, a separate entry will be provided for each country.
Income level of study setting	Lookup	Lookup in World Bank Country and Lending Groups using **Country of the Study**
Region of study setting	Lookup	Lookup in United Nations Geoscheme using **Country of the Study**
Study design	Dropdown	Select one from: RCT, Multiple case report, Case series, Cohort study, Cross sectional study, Retrospective study (unspecified), Prospective study (unspecified), or Other (Add as comment)
Number of infants with MMC-associated HC	Integer	This need not necessarily be the same as the sample size of the study (e.g., studies looking at non-MMC aetiologies of HC)

For peer review only

<table>
<tr><td>Age
(Mean, median, standard deviation and/or range)</td><td>Integers</td><td>In days. If multiple measures of central tendency or deviation present, extract all. If there are subgroups whose ages are within two years of birth, extract data for those subgroups. Do not extract data for any subgroups outside our age range. Regex validation string for range only: ^(\d|\d\d|[1-6]\d\d|7[0-2]\d|73[0-1])\.?(\d|\d\d)?-(\d|\d\d|[1-6]\d\d|7[0-2]\d|73[0-1])\.?(\d|\d\d)?$</td></tr>
<tr><td>Sex
(Female, male)</td><td>Integer</td><td>Of Number of infants with MMCaH.</td></tr>
<tr><td colspan="3">MMC management and outcomes</td></tr>
<tr><td>Signs or symptoms of MMC</td><td>Validated free text</td><td>Of Number of infants with MMCaH. Regex validation string: ^(.)+=(\s)?(\d|\d\d|\d\d\d|\d\d\d\d)((,(.)+=(\s)?(\d|\d\d|\d\d\d|\d\d\d\d))+)?$</td></tr>
<tr><td>Vertebral level of MMC
(Cervical, thoracic, thoracolumbar, lumbar, lumbosacral, sacral)</td><td>Integer</td><td>Of Number of infants with MMCaH. Compute as raw count if not given as such (e.g., if given as a proportion or percentage). Do not double count infants across vertebral levels.</td></tr>
<tr><td>Timing of MMC closure</td><td>Dropdown</td><td>Select one from: Ante-natal or post-natal. If mixed cohort, a separate entry will be provided for each closure.</td></tr>
<tr><td>MMC closure procedure and number of patients</td><td>Validated free text</td><td>The name of the intervention and the number of patients who had that intervention. Regex validation string: ^(.)+=(\s)?(\d|\d\d|\d\d\d|\d\d\d\d)((,(.)+=(\s)?(\d|\d\d|\d\d\d|\d\d\d\d))+)?$</td></tr>
</table>

For peer review only

<table>
<tr><td>Timing of MMC closure after birth</td><td>Validated free text</td><td>Groups/subgroups as reported by the authors. Regex validation string: ^(.)+=(\s)?(\d|\d\d|\d\d\d|\d\d\d\d)((,(.)+=(\s)?(\d|\d\d|\d\d\d|\d\d\d\d))+)?$</td></tr>
<tr><td>Age at MMC closure
(Mean, median, standard deviation and/or range)</td><td>Integers</td><td>As in Age. Regex validation string for range only: ^(\d|\d\d|\d\d\d|\d\d\d\d|\d\d\d\d\d)\.?(\d|\d\d)?-(\d|\d\d|\d\d\d|\d\d\d\d|\d\d\d\d\d)\.?(\d|\d\d)?$</td></tr>
<tr><td>Intra-operative mortality of MMC closure</td><td>Integer</td><td>Death during procedure. Compute as raw count if not given as such (e.g., if given as a proportion or percentage).</td></tr>
<tr><td>Peri-operative mortality of MMC closure</td><td>Integer</td><td>Death within 30 days of a procedure. Compute as raw count if not given as such (e.g., if given as a proportion or percentage).</td></tr>
<tr><td>Post-operative complications of MMC closure</td><td>Integer</td><td>Raw counts for Wound breakdown, Wound infection, and/or Meningitis. Regex validation string for miscellaneous only: ^(.)+=(\s)?(\d|\d\d|\d\d\d|\d\d\d\d)((,(.)+=(\s)?(\d|\d\d|\d\d\d|\d\d\d\d))+)?$</td></tr>
<tr><td colspan="3">First line intervention for HC</td></tr>
<tr><td>Patient sample</td><td>Dropdown</td><td>Select from: Selected or Unselected.</td></tr>
<tr><td>Age at diagnosis of HC
(Pre-natal or post-natal)</td><td>Integers</td><td>As in Age.</td></tr>
<tr><td>Method of diagnosis of HC
(CT imaging, MRI imaging, Ultrasonography, Clinical manifestation only, and Miscellaneous)</td><td>Integers</td><td>Raw counts for number of infants diagnosed with these methods. Regex validation string for miscellaneous only: ^(.)+=(\s)?(\d|\d\d|\d\d\d|\d\d\d\d)((,(.)+=(\s)?(\d|\d\d|\d\d\d|\d\d\d\d))+)?$</td></tr>
</table>

For peer review only

<table>
<tr><td>Indications for HC surgery
(Full or bulging fontanelle, Increased head circumference, CSF leaking from the wound, Symptoms of raised pressure, Other indications)</td><td>Integers</td><td>Raw counts for number of infants with these indications for HC surgery. Symptoms of raised pressure may include: These include: prominent scalp veins, high pitched cry, irritability, seizure, poor feeding, developmental delay, vomiting, setting sun sign, head suture and diastasis. Regex validation string for other indications only:
^(.)+=(\s)?(\d|\d\d|\d\d\d|\d\d\d\d)((,(.)+=(\s)?(\d|\d\d|\d\d\d|\d\d\d\d))+)?$</td></tr>
<tr><td>Co-morbidities
(Congenital heart defects, Congenital gastrointestinal malformations, Congenital face and neck malformations, Miscellaneous)</td><td>Integers</td><td>Raw counts for number of infants with these co-morbidities. Co-morbidities may not be recorded here if they are consequences of MMC/HC. Regex validation string for miscellaneous only:
^(.)+=(\s)?(\d|\d\d|\d\d\d|\d\d\d\d)((,(.)+=(\s)?(\d|\d\d|\d\d\d|\d\d\d\d))+)?$</td></tr>
<tr><td>Exacerbating factors
(Meningitis, Ventriculitis or Any other brain infection)</td><td>Integers</td><td>Raw counts for number of infants who had either meningitis or ventriculitis or any other brain infection before the HC.</td></tr>
<tr><td>Temporising procedures
(Subgaleal shunting or EVD)</td><td>Integers</td><td>Raw counts for number of infants who underwent subgaleal shunting or EVD as temporising procedures.</td></tr>
<tr><td>1st-line intervention for HC
(Conservative, VP shunting, ETV, Combined ETV and CPC, Failed ETV that was converted to VP shunt, or Miscellaneous)</td><td>Integers</td><td>Raw counts for number of infants who underwent one of these procedures as first-line treatment for HC. Non-surgical treatment is interpreted as conservative. If the infant underwent both VP and ETV, determine which was the primary one. For an infant to be recorded as a failed ETV that was converted to VP shunt,</td></tr>
</table>

For peer review only

<table>
<tr><td></td><td></td><td>the procedures must have happened in one sitting; alternatively, determine which was the primary one. Regex validation string for miscellaneous only: ^(.)+=(\s)?(\d|\d\d|\d\d\d|\d\d\d\d)((,(.)+=(\s)?(\d|\d\d|\d\d\d|\d\d\d\d))+)?$</td></tr>
<tr><td>Age at first-line procedure</td><td>Integers</td><td>As in Age.</td></tr>
<tr><td>Follow-up period</td><td>Integer</td><td>In weeks</td></tr>
<tr><td>Intra-operative mortality of 1st-line HC procedure</td><td>Integer</td><td>As in Intra-operative mortality of MMC closure</td></tr>
<tr><td>Peri-operative mortality of 1st-line HC procedure</td><td>Integer</td><td>As in Peri-operative mortality of MMC closure</td></tr>
<tr><td>Post-operative complications of 1st-line HC procedure (Wound, Procedure failure, Wound infection, Wound dehiscence, Meningitis, Ventriculitis, CSF leakage, Focal Neurological deficits, Overdrainage, Intracranial haemorrhage, Any biochemical disturbance, and Any other complications)</td><td>Integers</td><td>Raw counts for Wound, Procedure failure, Wound infection, Wound dehiscence, Meningitis, Ventriculitis, CSF leakage, Focal Neurological deficits, Overdrainage, Intracranial haemorrhage, Any biochemical disturbance, and Any other complications. Regex validation string for any biochemical disturbance and any other complications: ^(.)+=(\s)?(\d|\d\d|\d\d\d|\d\d\d\d)((,(.)+=(\s)?(\d|\d\d|\d\d\d|\d\d\d\d))+)?$</td></tr>
<tr><td>Number of procedures per patient</td><td>Integers</td><td>Raw counts.</td></tr>
</table>

For peer review only

(Mean, Median, Standard deviation, and/or Range)		
Second line intervention for HC		
Time to 1st procedure failure, if any (Mean, Median, Standard deviation and/or Range)	Integers	The time between 1st-line HC procedure and 2nd-line HC procedure. In weeks.
Any information on why the treatment failed	Free text	
2nd-line HC procedure	Integers	As in **1st-line HC procedure**
Age at second-line procedure	Integers	As in **Age at first-line procedure;** except that in weeks.
Intra-operative mortality of 2nd-line HC procedure	Integer	As in **Intra-operative mortality of 1st line HC procedure**
Peri-operative mortality of 2nd-line HC procedure	Integer	As in **Peri-operative mortality of 1st line HC procedure**
Post-operative complications of 2nd-line HC procedure	Integers	As in **Post-operative complications of 1st-line HC procedure**
Any further procedures for HC (repeat data items in **Second line intervention for HC** subheading)		
Overall outcomes		

Overall complication rate, if any (Mean, Median, Standard deviation, and/or Range)	Integers	Number of infants with any complications divided by the number of infants that underwent any surgery
Overall incidence rate, if any (Mean, Median, Standard deviation, and/or Range)	Integers	Number of procedure failures divided by the number of procedures. Also known as the overall failure rate

CPC: Choroid plexus cauterisation, ETV: Endoscopic third ventriculostomy, EVD: External ventricular drainage, HC: Hydrocephalus, MMC: Myelomeningocele, VP: ventriculoperitoneal

For peer review only

PRISMA-P 2015 Checklist

This checklist has been adapted for use with protocol submissions to *Systematic Reviews* from Table 3 in Moher D et al: Preferred reporting items for systematic review and meta-analysis protocols (PRISMA-P) 2015 statement. *Systematic Reviews* 2015 **4**:1

Section/topic	#	Checklist item	Information reported		Line number(s)
			Yes	No	
**ADMINISTRATIVE INFORMATION**					
**Title**					
Identification	1a	Identify the report as a protocol of a systematic review	☑	☐	1-3
Update	1b	If the protocol is for an update of a previous systematic review, identify as such	☐	☑	NA
**Registration**	2	If registered, provide the name of the registry (e.g., PROSPERO) and registration number in the Abstract	☑	☐	31
**Authors**					
Contact	3a	Provide name, institutional affiliation, and e-mail address of all protocol authors; provide physical mailing address of corresponding author	☑	☐	4-28
Contributions	3b	Describe contributions of protocol authors and identify the guarantor of the review	☑	☐	331-336
**Amendments**	4	If the protocol represents an amendment of a previously completed or published protocol, identify as such and list changes; otherwise, state plan for documenting important protocol amendments	☐	☑	NA
**Support**					
Sources	5a	Indicate sources of financial or other support for the review	☑	☐	346-347
Sponsor	5b	Provide name for the review funder and/or sponsor	☐	☑	NA
Role of sponsor/funder	5c	Describe roles of funder(s), sponsor(s), and/or institution(s), if any, in developing the protocol	☐	☑	NA
**INTRODUCTION**					
**Rationale**	6	Describe the rationale for the review in the context of what is already known	☑	☐	88-117
**Objectives**	7	Provide an explicit statement of the question(s) the review will address with reference to participants, interventions, comparators, and outcomes (PICO)	☑	☐	119-154

For peer review only

For peer review only - http://bmjopen.bmj.com/site/about/guidelines.xhtml

Section/topic	#	Checklist item	Information reported: Yes	Information reported: No	Line number(s)
**METHODS**					
**Eligibility criteria**	8	Specify the study characteristics (e.g., PICO, study design, setting, time frame) and report characteristics (e.g., years considered, language, publication status) to be used as criteria for eligibility for the review	☑	☐	191-247
**Information sources**	9	Describe all intended information sources (e.g., electronic databases, contact with study authors, trial registers, or other grey literature sources) with planned dates of coverage	☑	☐	181-189
**Search strategy**	10	Present draft of search strategy to be used for at least one electronic database, including planned limits, such that it could be repeated	☑	☐	181-189 and supplementary table 1
STUDY RECORDS					
Data management	11a	Describe the mechanism(s) that will be used to manage records and data throughout the review	☑	☐	249-251; 258-259; 278-284
Selection process	11b	State the process that will be used for selecting studies (e.g., two independent reviewers) through each phase of the review (i.e., screening, eligibility, and inclusion in meta-analysis)	☑	☐	249-255
Data collection process	11c	Describe planned method of extracting data from reports (e.g., piloting forms, done independently, in duplicate), any processes for obtaining and confirming data from investigators	☑	☐	257-268
**Data items**	12	List and define all variables for which data will be sought (e.g., PICO items, funding sources), any pre-planned data assumptions and simplifications	☑	☐	260 and supplementary table 2
**Outcomes and prioritization**	13	List and define all outcomes for which data will be sought, including prioritization of main and additional outcomes, with rationale	☑	☐	223-247
**Risk of bias in individual studies**	14	Describe anticipated methods for assessing risk of bias of individual studies, including whether this will be done at the outcome or study level, or both; state how this information will be used in data synthesis	☑	☐	270-276
DATA					
**Synthesis**	15a	Describe criteria under which study data will be quantitatively synthesized	☑	☐	286-308
	15b	If data are appropriate for quantitative synthesis, describe planned summary measures, methods of handling data, and methods of combining data from studies, including any planned exploration of consistency (e.g., I^2, Kendall's tau)	☑	☐	286-308
	15c	Describe any proposed additional analyses (e.g., sensitivity or subgroup analyses, meta-regression)	☑	☐	286-308
	15d	If quantitative synthesis is not appropriate, describe the type of summary planned	☐	☑	NA
**Meta-bias(es)**	16	Specify any planned assessment of meta-bias(es) (e.g., publication bias across studies, selective	☑	☐	286-308

For peer review only

Section/topic	#	Checklist item	Information reported		Line number(s)
			Yes	No	
		reporting within studies)			
**Confidence in cumulative evidence**	17	Describe how the strength of the body of evidence will be assessed (e.g., GRADE)	☑	☐	311-315

For peer review only

BioMed Central The Open Access Publisher
